# Shifting evolutionary sands: transcriptome characterization of the *Aptostichus atomarius* species complex

**DOI:** 10.1186/s12862-020-01606-7

**Published:** 2020-06-15

**Authors:** Nicole L. Garrison, Michael S. Brewer, Jason E. Bond

**Affiliations:** 1 School of Fisheries, Aquaculture, and Aquatic Sciences, Auburn University, 203 Swingle Hall, Auburn, AL 36849 USA; 2 Department of Biology, East Carolina University, Howell Science Complex N407, 1000 E 5th St, Greenville, NC 27858 USA; 3 Department of Entomology and Nematology, University of California Davis, Academic Surge Building 1282, Davis, CA 95616-5270 USA

**Keywords:** Mygalomorphae, Coastal dune, California Floristic Province, Transcriptomics, Species complex, Trapdoor spider

## Abstract

**Background:**

Mygalomorph spiders represent a diverse, yet understudied lineage for which genomic level data has only recently become accessible through high-throughput genomic and transcriptomic sequencing methods. The *Aptostichus atomarius* species complex (family Euctenizidae) includes two coastal dune endemic members, each with inland sister species – affording exploration of dune adaptation associated patterns at the transcriptomic level. We apply an RNAseq approach to examine gene family conservation across the species complex and test for patterns of positive selection along branches leading to dune endemic species.

**Results:**

An average of ~ 44,000 contigs were assembled for eight spiders representing dune (*n* = 2), inland (*n* = 4), and *atomarius* species complex outgroup taxa (*n* = 2). Transcriptomes were estimated to be 64% complete on average with 77 spider reference orthologs missing from all taxa. Over 18,000 orthologous gene clusters were identified within the *atomarius* complex members, > 5000 were detected in all species, and ~ 4700 were shared between species complex members and outgroup *Aptostichus* species. Gene family analysis with the FUSTr pipeline identified 47 gene families appearing to be under selection in the *atomarius* ingroup; four of the five top clusters include sequences strongly resembling other arthropod venom peptides. The COATS pipeline identified six gene clusters under positive selection on branches leading to dune species, three of which reflected the preferred species tree. Genes under selection were identified as Cytochrome P450 2c15 (also recovered in the FUSTr analysis), Niemann 2 Pick C1-like, and Kainate 2 isoform X1.

**Conclusions:**

We have generated eight draft transcriptomes for a closely related and ecologically diverse group of trapdoor spiders, identifying venom gene families potentially under selection across the *Aptostichus atomarius* complex and chemosensory-associated gene families under selection in dune endemic lineages.

## Background

**Fig. 1:**
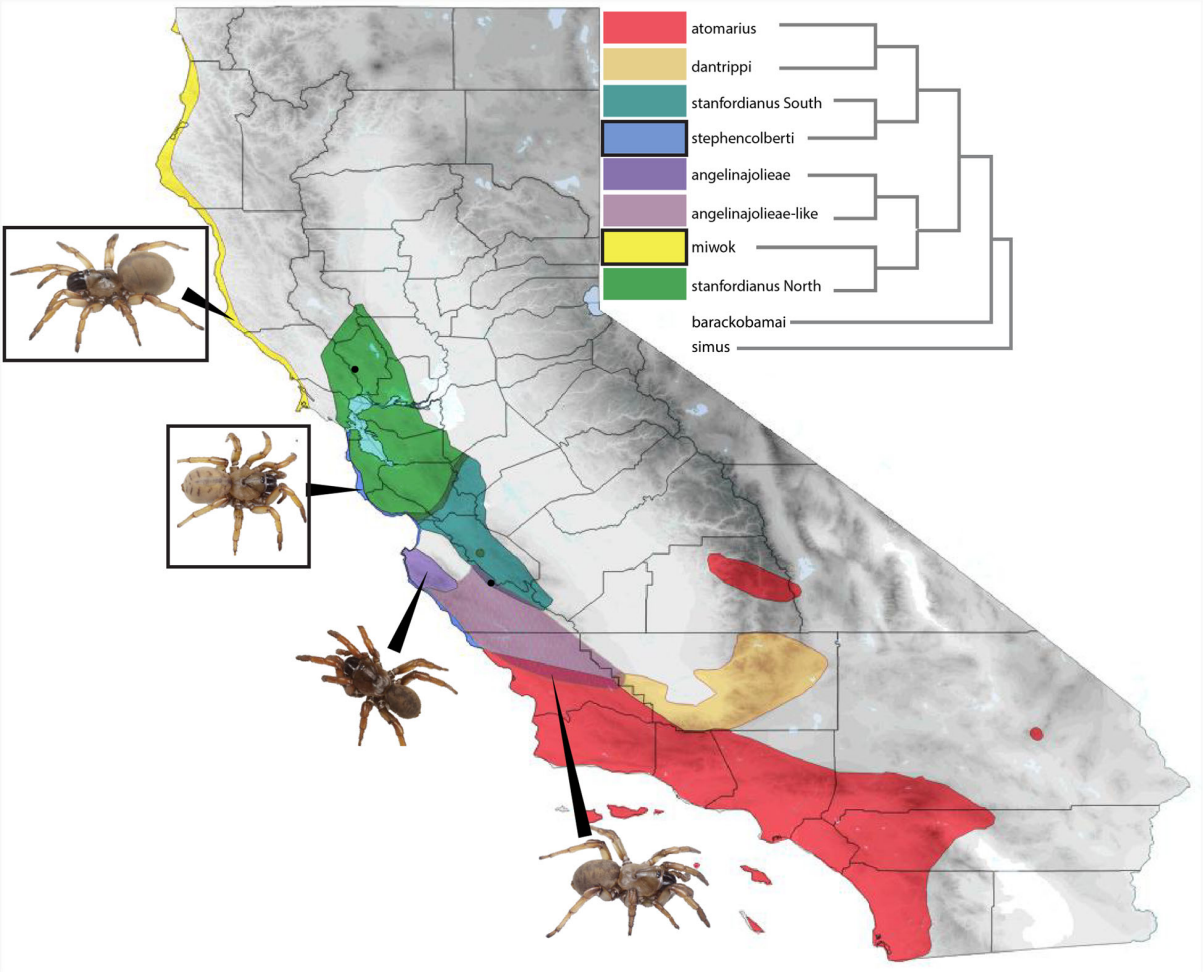
Generalized Distribution Map. Colors corresponding to the phylogenetic relationships depicted in the cladogram indicate general distributions of species in the *Aptostichus atomarius* complex, black dots and arrows indicate exact localities of individuals sampled. Boxes surround the names and pictures of dune endemic members of the species complex. Branch lengths of the cladogram do not represent evolutionary change and are only a representation of summarized phylogenetic relationships within this group of species

Trapdoor spiders belong to an ancient lineage of chelicerate arthropods, the spider infraorder Mygalomorphae, which includes charismatic fauna such as tarantulas and Australian funnel web spiders [1]. These spiders are sedentary, fossorial predators that build silk-lined burrows; females are non-vagile and mature males emerge seasonally to search for females. Mygalomorph spiders contain considerably less extant species diversity (348 genera, 3846 species) than their Araneomorph relatives (3732 genera, 44,534 species) [2], and have historically received less attention in the scientific literature. They present several challenges to researchers interested in performing rigorous experimental studies; they can be difficult to collect in large numbers from across their ranges, they are remarkably long-lived and take years to reach sexual maturity [3, 4], and, until recently, very few genetic markers and no genomic resources were available for the infraorder (but see [5, 6]). At the same time, they pose considerable appeal in terms of investigating physiological adaptation to harsh environments [7], longevity [8], evolution and application of novel venom peptides [9], chemosensory systems [10], genome size evolution [11], and historical biogeography, to name a few. With technological advances in sequencing, opportunities to begin generating genomic resources for non-model arthropods have increased substantially, from only three genomes in 2002 to over 540 at varying levels of completeness (27 at the chromosome level, 63 at the contig level, 458 at the scaffold level [12]). Even more accessible methods for non-model organisms such as phylogenomics, targeted genomic sequencing approaches, and comparative transcriptome efforts have begun to provide foundational data which may help resolve long-standing evolutionary questions and open new paths of inquiry for insects [13], spiders [14, 15], diplopods [16], and other arthropod groups [17]. Within mygalomorphs, second-generation sequencing approaches have recently been applied to the study of venoms [18], chemosensory systems [19], cryptic speciation [20], and higher-level systematics [21]. At the family level, publicly available sequence data for mygalomorph spiders has increased exponentially in the last five years due to large-scale phylogenomic analyses however; utilization of high throughput information to search for signatures of selection at the species level is terra incognita in mygalomorph research. The ability to carry out such studies at the species/population interface is hindered by a lack of appropriate foundational genomic datasets, as is the case for many non-model or ‘obscure model organisms’ [22]; only one mygalomorph spider genome has been partially sequenced, the tarantula *Acanthoscurria geniculata* (Koch, 1841), but remains in the scaffolding stage [5] and has likely been diverging from trapdoor spiders for ~114MY [14]. The overarching goal of this study is to build genomic resources and generate preliminary functional annotations for transcriptomes of an ecologically diverse trapdoor spider sister species complex. The *Aptostichus atomarius* complex is a closely related set of sister species pairs, a sibling species complex, distributed throughout the Coastal Ranges in the California Floristic Province. Of the seven members, two species are chaparral dwelling, two are coastal dune endemics, and three inhabit the inland hills and valleys of central California west of the Central Valley [23]. The two dune species represent independent colonization of dune habitats, and though they share phenotypic features of light pigmentation and reduced abdominal patterning [24], they are not sister taxa (Garrison et al. 2019, unpublished in prep). *Aptostichus miwok* occupies dune habitats north of the San Francisco Bay and *A. stephencolberti* is distributed along beaches further to the south (Fig. 1). We have utilized RNAseq derived sequences to generate draft transcriptome assemblies, annotations, and search for gene families under selection within the *A. atomarius* complex; we specifically test for positive selection in detected orthologs along branches of the species tree leading to dune endemic members. We also assess transcriptome level conservation across the complex and between *A. atomarius* members and two outgroup *Aptostichus* species representing varying levels of taxonomic distance from the species complex ingroup. For clarity, species included in the *atomarius* ingroup (*n* = 6) are *A. atomarius*, *A. miwok* (dune endemic), *A. stephencolberti* (dune endemic), *A. angelinajolieae*, and *A. stanfordianus* (North and South clades). The outgroup (*n* = 2) includes *A. simus* and *A. barackobamai*.

## Results & discussion

### Sequencing and data processing

Raw read counts ranged from ~ 27 to 61 million paired reads, averaging ~ 29 million for the 25 M read sequencing design (*A. atomarius*, *A. angelinajolieae*, *A. miwok*, *A. stanfordianus* North, and *A. stephencolberti*) and ~ 49 million for the 50 M design (*A. stanfordianus* South, *A. barackobamai*, *A. simus*). Mean base quality scores as assessed by FastQC were > 30 for all raw reads, however, post sequencing Illumina adapter contamination was detected and removed using Trimmomatic during assembly. Pre- and post-assembly statistics for each transcriptome can be found in Table 1; total number of assembled contiguous sequences (contigs) ranged from 30,871–61,516 with a mean length of 636 and average GC content of 40%. *A. stephencolberti* had the fewest contigs (30,871), while *A. stanfordianus* North had the most (61,516). On average, there were ~ 35,700 unique genes with isoform group size ranging from 2 to 38. Isoform distribution was less expansive for earlier sequencing events (25 M PE samples), group size decreased drastically for all assemblies beyond the 3-isoform category.

**Table 1:** Sample Summary and Metadata Transcriptome pre and post sequencing summaries and associated metadata

Sample	MY4009	AUMS62	AUMS20	AUMS29	AUMS33	AUMS20723	AUMS01	AUMS22
Species	atomarius	angelinajolieae	stephencolberti	miwok	stanfordianus North	stanfordianus South	barackobamai	simus
Design	25 M,PE	25 M,PE	25 M,PE	25 M,PE	25 M,PE	50 M,PE	50 M,PE	50 M,PE
Read #	27,431,535	30,880,739	30,904,990	28,351,749	28,168,870	67,199,206	58,216,062	50,721,762
Read Len	50	50	50	50	50	50	50	50
Contigs	35,444	46,796	30,871	47,390	61,516	50,708	43,524	36,628
Genes	34,781	45,664	30,227	30,340	58,265	38,912	34,635	30,340
ORFs	14,714	17,997	14,717	18,348	21,229	23,075	21,709	18,577
UniProt (blastx)	11,432	13,499	11,519	13,397	16,348	15,645	15,100	13,296
UniProt (blastp)	8226	9880	8415	9905	11,546	10,996	10,801	9334
Tarantula	17,186	20,201	16,804	20,307	25,580	23,724	22,085	19,607
UniRef90	14,257	17,186	14,300	17,177	21,334	20,207	19,118	16,836
lat	35.41695	36.571374	36.704522	38.307402	38.417361	36.432667	38.70425	36.704522
long	−120.55722	−121.904289	−121.803911	− 123.053548	− 122.662169	−121.228455	−122.93653	− 121.803911

RSEM mapping rates prior to de-duplication ranged from 71.7–86.6%, with larger more isoform rich transcriptomes averaging 72% and less diverse assemblies averaging 84%. Assessment of completeness via TransRate resulted in ‘good’ sequence files containing ~ 17,260 contigs on average. Mapping rates determined by the TransRate pipeline were lower than those generated via RSEM with an average mapping rate of 66% and ‘good’ mapping rates averaging 58%. A critical step in understanding the patterns of transcriptional diversity within this species complex is establishing confidence in species identifications. This group of morphologically indistinguishable spiders have been shown previously to display significant levels of mitochondrial divergence [23, 24], therefore we utilized publicly available mitochondrial sequences for the complex to confirm the identity of individuals sampled in transcriptome analyses. Mitochondrial matching of samples to previously sequenced localities was successful in all but two cases: *A. atomarius* and *A. stanfordianus* South may represent a previously unrecognized clade of *Aptostichus* occurring south of the *A. angelinajolieae* range (see Fig. 1, angelinajolieae-like). This clade was found to be sister to *A. angelinajolieae* in the recent revision of the genus but was not explicitly analyzed in the species tree analyses of Garrison et al. (2019, unpublished in prep). Importantly, the identity of the dune endemic species sampled – *A. miwok* and *A. stephencolberti* – is unquestionably correct and therefore does not impair our ability to compare dune lineages with their inland relatives. Original species names have been retained for the purposes of this study, pending further examination of speciation within the complex.

**Fig. 2:**
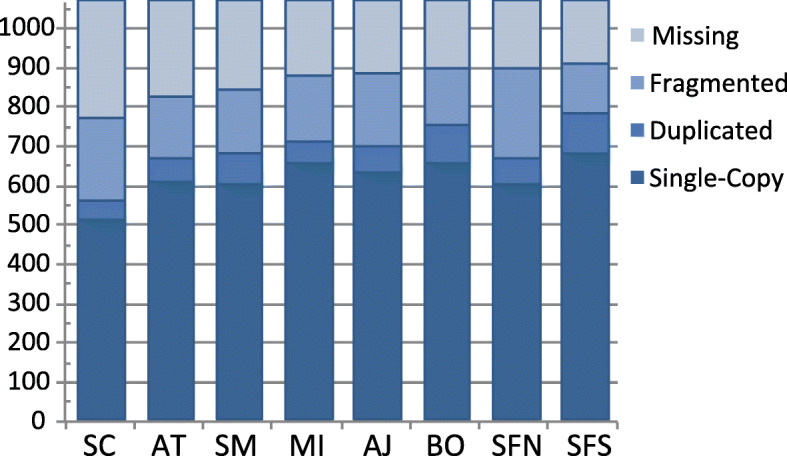
BUSCO Results. Bars represent the number of missing, fragmented, duplicated, and single-copy orthologs detected for each species sampled relative to *Parasteatoda* reference orthologs. Abbreviations correspond to specific epithets – *stephencolberti* (SC), *atomarius* (AT), *simus* (SM), *miwok* (MI), *angelinajolieae* (AJ), *barackobamai* (BO), *stanfordianus* North (SFN, and *stanfordianus* South (SFS)

Completeness as assessed by BUSCO [25] showed that *Aptostichus* transcriptomes were ~ 64% complete when compared to the *Parasteatoda* reference sequences (Fig. 2). The smallest transcriptome, *A. stephencolberti* was the least complete (52%) while *A. stanfordianus* South was the most (72%). Of the genes missing, 77 were missing from all of the *Aptostichus* transcriptomes. Missing sequences were found to represent five functional annotation clusters by the online functional annotation tool DAVID. Two KEGG pathways were identified, having multiple components missing – the Fanconi anemia and glycerophospholipid metabolism pathways. The Fanconi anemia pathway comprises 19 core and other associated proteins which carry out many different cellular functions including DNA repair, ensuring efficient DNA replication, maintaining telomere length, and selective autophagy. Absence, reduction, or modification of this pathway has serious repercussions in mammals causing growth of tumors, congenital defects, and bone marrow failure [26] . FANCD1, a core protein in the pathway, is an alternate name for the breast cancer associated gene BRCA2 [27]. The role and extent of this pathway outside of mammals is not well studied; however, a handful of Fanconi pathway orthologs have been detected in model invertebrates [28] and a reduction in the number of components has been uncovered in the simple chordate *Ciona intestinalis* [29, 30]. The dearth of pathway components in *Aptostichus* may be an indication that Mygalomorph spiders have a modified Fanconi pathway relative to the araneomorph spider used as a reference. It is possible that the full pathway was not being expressed in any of the spiders sampled, but due to the number of individuals and diversity of tissue sampled in this study further investigation is warranted. Only sixteen Fanconi anemia related genes are present in the OrthoDB at the level of Arachnida, and only 8 of those are present in the reference taxon used in our analyses (https://www.orthodb.org/?level=6893&species=114398_0&query=Fanconi). The missingness of this pathway and its components is a much larger question which must be addressed at multiple taxonomic levels; database bias is likely a large component in our inability to detect fully assembled transcripts of these proteins and does not in any way exclude the possibility that the pathway exists in Mygalomorph spiders.

### Ortholog detection

**Fig. 3:**
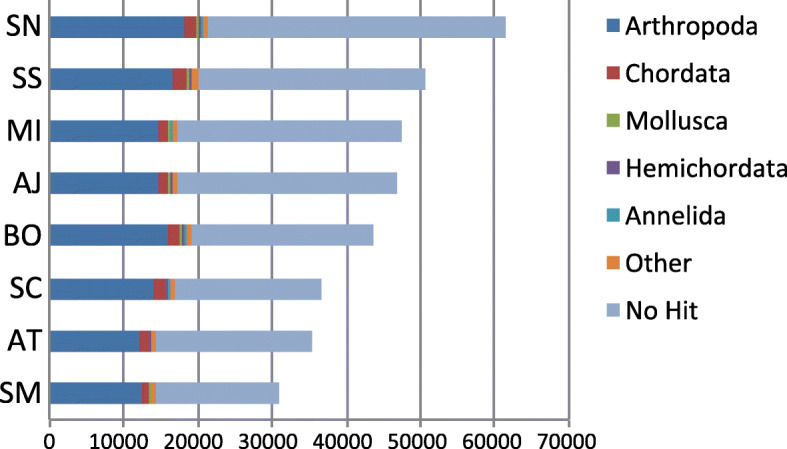
Taxonomic Affiliations. Taxonomic assignments of contigs for each species as determined by MCSC. Abbreviations are as in Fig. 2

**Fig. 4:**
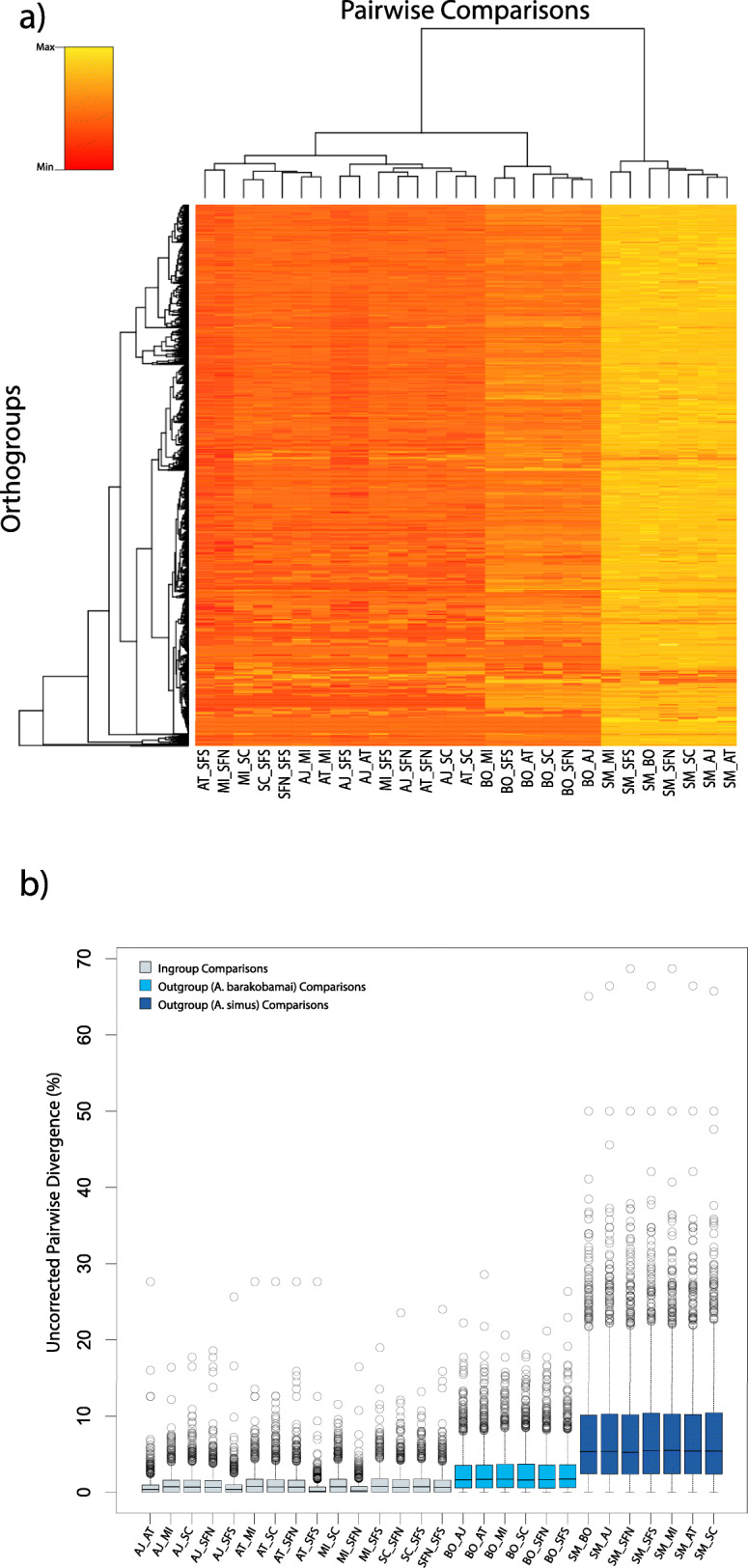
Uncorrected Pairwise Genetic Differences **a)** Hierarchical heatmap of uncorrected pairwise distances for each species comparison and detected orthogroup **b)** Boxplot visualization of uncorrected pairwise divergences showing quantitative differences in magnitude of divergence within the *atomarius* complex and between ingroup members and the two outgroup species

Decontamination with MCSC revealed high taxonomic affinity with Arthropoda for sequences that had matches to the uniref90 database; however, most transcripts had no similarity to sequences in the database (Fig. 3). Despite this, MCSC recovered ~ 27,247 sequences on average which passed the taxonomy/clustering filter. The full complement of transcripts was processed with OrthoFinder and high confidence sequences representing overlap between MCSC and TransRate were processed with OrthoVenn, generating a rich resource of orthologous clusters for species level comparisons. For the atomarius complex ingroup, OrthoFinder assigned 96,946 genes (88.1% of total) to 18,273 orthogroups. Fifty percent of all genes were in orthogroups with 6 or more genes (G50 was 6) and were contained in the largest 6577 orthogroups (O50 was 6577). There were 5770 orthogroups with all species present and 2127 of these consisted entirely of single copy genes. When the outgroup taxa were compared as well, OrthoFinder assigned 134,045 genes (89.1% of total) to 19,773 orthogroups. Fifty percent of all genes were found in orthogroups with 8 or more genes (G50 = 8) and were contained in the largest 6230 orthogroups (O50 = 6230). There were 4799 orthogroups with all species present and 1338 of these consisted entirely of single-copy genes. Uncorrected pairwise distances were calculated for alignments of single copy orthogroups recovered in the OrthoFinder analysis including outgroups (*n* = 1338) using the EMBOSS utility distmat [30–32] and visualized using R (Fig. 4a). The minimum uncorrected distance estimated for all orthogroups was 0, 35 groups were highly conserved across all taxa and 47 were conserved across all ingroup taxa. Maximum pairwise divergence estimated was 68.7%., however values in this range were only detected in *A. simus*/ingroup pairwise comparisons and are statistical outliers (Fig. 4b).

**Fig. 5:**
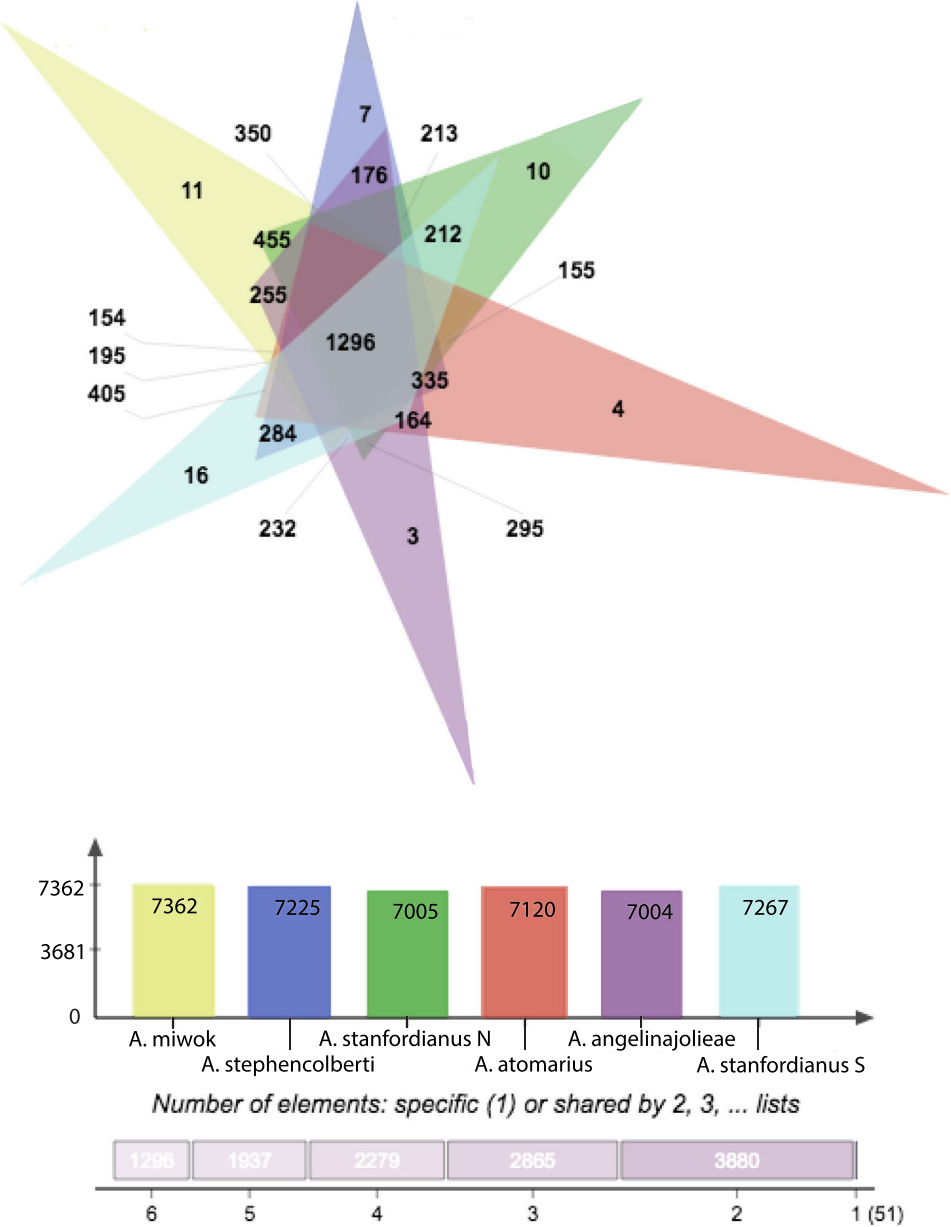
OrthoVenn Results. OrthoVenn generated visualization of ortholog overlap between species in the *atomarius* complex ingroup (above) and total number of orthologs included in the analysis for each species (below)

Pairwise differences between ingroup members were less than 1% on average (excluding the conserved orthogroups). Differences were particularly low in *A. atomarius*/*A. angelinajolieae*/*A. stanfordianus* South comparisons. and between *A. miwok* and its inland sister species *A. stanfordianus* North. Average pairwise divergence between all ingroup taxa and *A. simus* was around 5.3% and about 1.7% between ingroup taxa and *A. barackobamai*. In total, the high confidence filtering of transcripts with OrthoVenn yielded 1296 orthogroup clusters with representative sequences from all species; more species-specific clusters were detected with this method, and there were only 717 single copy gene clusters (Fig. 5).

### Gene families under selection

FUSTr detected 46 gene families under some degree of positive selection (Additional file 1) within the atomarius complex ingroup, with the number of sites under selection ranging from 1 (*n* = 26) to 18 (*n* = 1). Four of the five top clusters under selection were composed of venom related peptides. The cluster of orthologs with the most sites under selection shared significant homology with the ICK (inhibitor cysteine knot) protein family, a group of hyperstable small peptides which have been detected in most spider venom proteomes [33]. The specific peptides detected in *Aptostichus* most closely resemble the Aptotoxins (a.k.a. Cyrtautoxins [31];), isolated from the mygalomorph spider *Apomastus schlingeri* [34] with BLAST identities ranging from 42 to 59%. When the *Aptostichus* ICK peptide structure was compared to the PDB database, it was found to most resemble U4-hexatoxin-Hi1a with a very high TM-align score of 0.962. Not only do these venoms act as strong paralytic insecticides, they are remarkably resistant to proteases and environmental degradation (extreme pH, organic solvents, temperature extremes) making them candidates for orally active therapeutics [35] The cluster with the second highest number of sites under selection belonged to the Kunitz family of venom peptides, which are serine protease inhibitors (ArachnoServer [31];). Other venom peptides detected in the top 20 families under selection included Techylectin-like homologs (agglutinate in human erythrocytes and Gram+/− bacteria), and Prokinektin-2-like proteins (CsTx-20, neurotoxic enhancer). The cluster with the third highest number of sites under selection was an alphatocopherol (vitamin E) transferase family, with 8 sites under strong positive selection. Only two families were found to be under selection in the dune endemic spiders retinol dehydrogenase and Cytochrome P450. Both of these families were also detected in the complete ingroup analysis as well; this is not likely a dune endemic specific result. The COATS pipeline revealed six orthologous clusters under strong positive selection that met the 0.05 FDR (false discovery rate) threshold cutoff. Three of these groups matched the input species tree topology. Among the three groups with the appropriate species tree topology (Additional file 2) were Cytochrome P450 2c15 (as in the FUSTr analysis), Niemann Pick C1-like, and Kainate 2 isoform X1 (ionotropic glutamate receptor) as identified by NCBI-BLAST. Both Niemann Pick and Kainate/glutamate receptor sequences were detected in a recent distal leg tissue specific transcriptome analysis of the mygalomorph spider *Macrothele calpeiana* and may play a role in chemosensory function [19]. *Aptostichus* sequences display strong similarity (64–85% pairwise identity) at the nucleotide level to four of the six chemoreception candidate genes identified from leg tissue in that study (2 Niemann Pick C2 and 2 glutamate receptor genes). The COATS pipeline detected selection in a few proteins belonging to families with some venom associations sulfotransferase, A-disintegrin and metalloproteinase with thrombospondin motif (ADAMTs5), and even cytochrome p450 is a large gene superfamily involved in electron transport chains and has been implicated in many physiological pathways.

### Coastal dune evolutionary context

The California coastline is constantly changing shape, transformed by the action of wind, waves, and sun yet maintaining a characteristic succession of dune habitats due to specific constraints imposed by those same forces [36]. As in many arid and dune habitats, the organisms which occupy this type of environment often display a similar psammophilic evolutionary syndrome. Adaptation trends toward conservation of moisture, avoiding or dealing with increased sun exposure, and altered morphologies to contend with sand [37]. To a colonizing organism, a coastal dune habitat presents many abiotic and biotic challenges that differ from inland habitats which, over evolutionary timescales, might result in signals of selective pressure. Drought, disturbance, and the unique chemical composition of dune soils have led to the development of specific community structures in sand dune ecosystems particularly across the dune-inland gradient [38]. Implications of *Aptostichus* dune colonization may include higher levels of oxidative stress from temperature extremes, increased salinity, and a decrease in soil moisture requiring or resulting in altered metabolic responses. Due to the unique arthropod community of coastal dunes [39], a top arthropod predator would encounter a diet that is divergent in species composition from inland habitats. This trophic shift might result in a divergent venom profile. Altered macro and micronutrient availability, changes in the microbiome or composition of burrow associated soil bacteria/fungi, and engineering challenges associated with constructing and maintaining a burrow in shifting sand would result in a modified burrow environment. And finally, an altered signaling landscape due to substrate and vegetation changes could result in behavioral modification to male search strategies or chemical communication between spiders. This work provides a foundation for future studies of the connection between speciation, genome-wide divergence, and adaptation to coastal dune habitats. The changes in phenotype observed in dune spider lineages represent the shallowest level of response to dune colonization; we detected positive selection at the amino acid level for genes related to venom production, metabolism, and sensory systems. The complexity of both the habitat and transcriptional patterns will require much more fine-scale analysis to make strong connections between ecology and species-specific adaptations.

### Future directions

Guided by this study, areas of future research interest include specific differences in composition and nutritional content of diet, abiotic dune parameters, and secretion of volatile compounds which might be associated with inter or intra species signaling in *Aptostichus*. Additionally, tissue specific and transcriptomes sampled from males may be very revealing in this group – increasing resolution and specificity of datasets will make inferring function easier. Examining males, with their reduced life span, altered phenotype, and epigean life stage, would provide a more complete picture of dune adaptation in these species. To extract the maximal amount of insight from resources like those generated in this study, complementary natural history studies are critical. What are the spiders eating? When do they move across the landscape and why? How are they communicating, what kinds of interactions are they having with each other? Are there species-specific and/or habitat related parasitoid pressures that might differently impact population dynamics and chemical communication? More detailed knowledge of the environmental constraints imposed upon these spiders and their associated life history strategies will guide future work and provide a more complete context for the results of the current study.

## Conclusions

This work sets the stage for further comparative studies using this species complex as a model system for investigating the physiological, behavioral, and genetic consequences of adaptation to novel arid habitats. Understanding why and how these species diverged and the specific adaptations they developed to cope with an ever-changing landscape will be informative outside of California’s coastal habitats. Climate change will mean an increase in transitions to arid environments for many species at a far more rapid pace than previously experienced. Our understanding of how those transitions occur and what determines the success of dune colonization is currently limited. The transcriptome assemblies presented here also represent a novel genomic resource for researchers interested in spider/chelicerate evolution or species level variation in transcription. We have developed a first transcript level reference of shared orthologs for a closely related set of mygalomorph spiders, detected genes under putative positive selection in independent colonizers of dune habitats, and recovered gene families containing novel peptides across the atomarius complex. Mygalomorphs harbor a vast amount of evolutionary insight regarding early animal evolution, physiology, and synthesis of potent chemical cocktails. This study opens up many novel avenues of research within *Aptostichus* and for other dune inhabiting species.

## Methods

Adult female spiders were collected from known localities with mitochondrial evidence for clade assignment [23] for five of the six currently recognized species in the atomarius complex (*A. atomarius*, *A. angelinajolieae*, *A. stephencolberti*, *A. miwok*, and *A. stanfordianus* North); one individual from the putative cryptic species *A. stanfordianus* South was also obtained. Two outgroup taxa, *A. barackobamai* and *A. simus*, w ere also sampled for this study. After burrow excavation, all spiders were placed in individual containers with sterile tissue wipers molded into a burrow shape, transported back to the lab, and held for two weeks under the same conditions (room temperature, minimal light exposure, daily hydration, no food). After a multi week holding period, spiders were removed from their artificial burrows and flash frozen in preparation for RNA extraction. The prosomal region of each spider was cut diagonally in half and, with the distal portion of one leg, was ground in liquid nitrogen before being transferred to a tube containing 1 mL TRIzol. RNA was extracted following the TRIzol protocol with an additional RNA purification step using the RNeasy kit (Qiagen). Samples were checked for high quality via spectrometry and gel electrophoresis and sent to the Genomic Services Center at HudsonAlpha (Huntsville, Alabama) for paired end sequencing on the Illumina HiSeq platform (50 bp, 25–50 million reads). Collection and processing of spiders in this study happened in three pulses – sequencing details, raw sequence statistics, and locality information for each specimen is summarized in Table 1.

### Assembly and assessment of completeness

Raw sequence reads were processed with the program FastQC to evaluate sequence quality and content. Guided by the FastQC results, residual Illumina adapters were removed with Trimmomatic [40] during assembly. The program Trinity v2.2.0 [41, 42] was used to generate de novo assemblies for each of the individuals, using default paired end parameters. To estimate assembly statistics and provide expression level data for downstream interpretation of functional annotations, raw reads were mapped back to their respective assemblies using the programs RSEM [43] and TransRate [44]. PCR duplicates were removed from raw reads using samtools rmdup [45] prior to final mapping to references to ensure more accurate coverage estimation. TransRate uses the ultrafast alignment algorithm of SNAP (Scalable Nucleotide Alignment Program) [46] to map reads back to transcriptomes and the alignment-free mapping software salmon [47] to assign multi mapping reads and generate coverage values. TransRate generates a filtered subset of contigs based on read coverage evidence as well as descriptive statistics about each assembly. After assembly, 12S-tRNA Val-16 s mitochondrial fragments were extracted and used to match samples to previously sequenced haplotypes and confirm species identities. BUSCOv3 (Benchmarking Universal Single-Copy Orthologs [25, 48];) was used to determine completeness of the assembly relative to a curated, highly conserved set of single-copy orthologs housed in the OrthoDB online database. The BUSCO pipeline first translates and detects open reading frames (ORFs) within a set transcriptome contigs (using TransDecoder; http://transdecoder.github.io), then uses hidden markov models (HMMER [49];) to search the curated ortholog set for matches, accepting those sequences which are recovered as reciprocal best hits to the reference species of choice. For this study BUSCO was used to determine the proportion and quality (complete, fragmented, duplicated) of 2675 core arthropod (fly reference species) and 1066 core spider (*Parasteatoda* reference) orthologs present in each transcriptome. BUSCO analyses were executed on the CyVerse Discovery Platform (www.cyverse.org) for all species. The transcriptomes were further evaluated for taxonomic identity of sequence clusters using MCSC decontamination [50]. MCSC uses hierarchical clustering approach and incorporates taxonomic information from BLAST [51] hits to the UniRef90 cluster database to determine which sequences likely represent the focal organism and which may represent contaminating organisms. Contamination can arise from sources within and on the surface of the extracted tissues or potentially during sample/library preparation and sequencing via sample bleeding [52]. Though the expectation is minimal contamination given the tissue types chosen, MCSC was used to exclude transcripts with no homology to known spider or arthropod transcripts in the final set of contigs. MCSC was employed at the phylum level; Arthropoda best hits were preferentially retained. Taxonomic distributions based on BLAST hits for each of the species were parsed from the MCSC results and ‘good’ transcripts represented in both the MCSC and TransRate filtered files were used for downstream ortholog inference.

### Functional annotation

Annotations were added to the full set of transcripts for each species using the Trinotate pipeline. First, untranslated transcriptome sequences and predicted open reading frames for each species were subjected BLAST+ [53] searches of the UniProt peptide database (blastx and blastp respectively). Additional blastp and blastx searches were conducted using proteins predicted from the reference tarantula transcriptome [6] as a database. Next, HMMER was used to search for protein family domains using the PfamA database [54], signalP [55] was used to search for signal peptide cleavage sites, tmHMM [56] was used to identify transmembrane regions, and RNAmmer [57] was used to detect any ribosomal RNA present in the samples. Trinotate output includes eggnog [58] and KEGG [59] associated terms for all annotated contigs when able. All results were loaded into a boilerplate sqlite database before being exported into a tab-delimited report that could be parsed in downstream analyses. OrthoFinder [60] and the online ortholog visualization tool OrthoVenn [61] were used to identify and compare sets of orthologs across the *Aptostichus* samples and within the atomarius ingroup. OrthoFinder offers improved accuracy and recovery relative to several other ortholog detection programs by overcoming sequence length biases in ortholog detection [60]. The full complement of coding sequences predicted from each transcriptome and the filtered set (TransRate/MCSC overlap) was processed with OrthoFinder to determine orthogroup overlap and identify species-specific orthogroups. OrthoVenn is an online orthology server which combines OrthoMCL, BLAST homology searches of the swissprot reference database, and inparalog detection with orthAgogue [62] to generate interactive visualizations of whole genome/transcriptome comparisons. In OrthoVenn, the filtered and translated transcripts were analyzed for the full *A. atomarius* complex ingroup.

### Detection of gene families under selection

The FUSTr pipeline (Families Under Seletction in Transcriptomes [63];) was used to explore patterns of selection 1) within the atomarius complex and 2) within dune endemic species. For detection of genes under selection, the full set of transcripts was utilized for each species under the expectation that rare or lowly expressed transcripts may contribute to a pattern of gene family expression in a biologically meaningful way. This approach provides the maximum amount of transcriptome wide information while still allowing for incorporation of confidence estimates from TransRate, MCSC, and RSEM in post-analysis interpretation of findings if necessary. FUSTr first translates sequences and predicts open reading frames (Trans-Decoder), infers homology using blastp and the transitive clustering algorithm of SiLix [64], generates multiple sequence alignments of clusters using mafft [65], and builds phylogenetic trees for each family using FastTree [66] prior to detection of selection. In families containing at least 15 members, site-specific tests for positive selection (amino acid level) are performed using codeml v4.9 [67] and log likelihood values are compared to those of models excluding positive selection. The result of FUSTr analysis is a list of gene families detected, and a file highlighting those containing at least one site where the ratio of non-synonymous to synonymous changes (dN/dS ratios, ω) exceeded 1, indicating strong positive selection. Tests for positive selection along branches leading to dune endemic species *A. miwok* and *A. stephencolberti* were implemented using the COATS pipeline (unpublished, Brewer et al. in prep, https://hub.docker.com/r/michaelsbrewer/coatstest), which is designed to examine selection within the context of a species tree. The species tree generated with the most corroboration across analyses (Fig. 1, legend) was given to the pipeline for the multi-species analysis pathway. Briefly, TransDecoder predicted ORFs are subjected to an all versus all blastp search, reciprocal best hit loci are used to generate fasta files with orthologous sets of loci, orthologous sets are searched using a reference taxon (in our case the dune species *A. stephencolberti*), orthologs are aligned using mafft, pal2nal.pl [68] is used to assign codon positions to sequences using the translated ORF and corresponding nucleotide sequences, poorly aligned sites in alignments are masked using Aliscore/Alicut [69] alignments with too few taxa are removed, and multi-species PAML [67] analyses are performed on the remaining alignments.

## Supplementary information

**Additional file 1.** List of gene families under selection and number of sites under selection in each, top blast hit identities of gene families.

**Additional file 2.** Gene families ordered by strength of selection detected by the COATS pipeline. Identities of genes showing both positive selection and having an assignable identity are highlighted.

## Data Availability

*Aptostichus atomarius* (SRS3660119) and *Aptostichus stephencolberti* (SRS3660108) sequence reads are available on the Short Read Archive. The datasets supporting the conclusions of this article (assembled transcriptomes, annotations) are available in the AptostichusAssemblies repository, https://github.com/nlgarrison/AptostichusAssemblies.

## Abbreviations

RNAseq: Ribosomal nucleic acid sequencing
FUSTr: Families under selection in transcriptomes
MY: Million years
ICK: Inhibitor cysteine knot
BLAST: Basic local alignment search tool
SNAP: Scalable nucleotide alignment program
FDR: False discovery rate
ORF: Open reading frame
